# Malaria Rapid Diagnostic Tests and Malaria Microscopy for Guiding Malaria Treatment of Uncomplicated Fevers in Nigeria and Prereferral Cases in 3 African Countries

**DOI:** 10.1093/cid/ciw628

**Published:** 2016-12-06

**Authors:** Catherine O. Falade, IkeOluwapo O. Ajayi, Jesca Nsungwa-Sabiiti, Mohamadou Siribié, Amidou Diarra, Luc Sermé, Chinenye Afonne, Oyindamola B. Yusuf, Zakaria Gansane, Ayodele S. Jegede, Jan Singlovic, Melba Gomes

**Affiliations:** 1 Department of Pharmacology and Therapeutics; 2 Department of Epidemiology and Medical Statistics, College of Medicine, University of Ibadan, Nigeria; 3 Child Health Division, Ministry of Health, Kampala, Uganda; 4 Groupe de Recherche Action en Santé, Ouagadougou, Burkina Faso; 5 Epidemiology and Biostatistics Research Unit, Institute of Advanced Medical Research and Training, College of Medicine; 6 Department of Sociology, Faculty of Social Sciences, University of Ibadan, Nigeria; 7 UNICEF/UNDP/World Bank/WHO/Special Programme for Research & Training in Tropical Diseases, World Health Organization, Geneva, Switzerland

**Keywords:** malaria, microscopy, RDT, ACT, parasitemia

## Abstract

***Background.*** The World Health Organization recommends that malaria treatment be based on demonstration of the infecting *Plasmodium* parasite specie. Malaria rapid diagnostic tests (RDTs) are recommended at community points of care because they are accurate and rapid. We report on parasitological results in a malaria study in selected rural communities in 3 African countries.

***Methods.*** In Nigeria, community health workers (CHWs) performed RDTs (SD-Bioline) and thick blood smears on all children suspected to have malaria. Malaria RDT-positive children able to swallow received artemisinin-based combination therapy (Coartem). In all countries, children unable to take oral drugs received prereferral rectal artesunate irrespective of RDT result and were referred to the nearest health facility. Thick blood smears and RDTs were usually taken at hospital admission. In Nigeria and Burkina Faso, RDT cassettes and blood smears were re-read by an experienced investigator at study end.

***Results.*** Trained CHWs enrolled 2148 children in Nigeria. Complete parasitological data of 1860 (86.6%) enrollees were analyzed. The mean age of enrollees was 30.4 ± 15.7 months. The prevalence of malaria parasitemia in the study population was 77.8% (1447/1860), 77.6% (1439/1855), and 54.1% (862/1593) by RDT performed by CHWs vs an expert clinical research assistant vs microscopy (gold standard), respectively. Geometric mean parasite density was 6946/µL (range, 40–436 450/µL). There were 49 cases of RDT false-negative results with a parasite density range of 40–54 059/µL. False-negative RDT results with high parasitemia could be due to non-falciparum infection or result from a prozone effect. Sensitivity and specificity of SD-Bioline RDT results as read by CHWs were 94.3% and 41.6%, respectively, while the negative and positive predictive values were 86.1% and 65.6%, respectively. The level of agreement in RDT reading by the CHWs and experienced research staff was 86.04% and κ statistic of 0.60. The malaria parasite positivity rate by RDT and microscopy among children with danger signs in the 3 countries was 67.9% and 41.8%, respectively.

***Conclusions.*** RDTs are useful in guiding malaria management and were successfully used for diagnosis by trained CHWs. However, false-negative RDT results were identified and can undermine confidence in results and control efforts.

Malaria exerts an unacceptably high toll on children in Africa, where 80% of estimated malaria cases and 90% of deaths occur, especially in Nigeria [1]. Early diagnosis and prompt, effective treatment are recommended in malaria control guidelines [2]. However, most cases of malaria in Africa are still diagnosed presumptively [3], with consequent overdiagnosis of malaria because the symptoms and signs of malaria are generally nonspecific.

There have been major changes in recommendations for malaria management in order to reduce morbidity and mortality [2, 4, 5]. The World Health Organization (WHO) recommends that treatment of acute uncomplicated malaria be based on artemisinin-based combination therapy (ACT) [1, 5] with rectal artesunate as prereferral treatment of severe malaria [5] before transit to a hospital. Furthermore, WHO recommends that all malaria case management be based on parasitological diagnosis [5], a policy that was adopted by the Nigerian National Malaria Programme in 2011 [6]. The recommended parasitological tests are light microscopy and immunochromatographic rapid diagnostic tests (RDTs). Microscopy of Giemsa-stained blood smears remains the gold standard for confirmation of malaria diagnosis. Microscopy has numerous advantages. It allows identification and quantitation of the causative organism. It is also cheap and excellent in competent hands. However, light microscopy is not a feasible option in most parts of sub-Saharan Africa because of irregular electricity to power microscopes that are in short supply. In addition, suitably trained laboratory technicians are not generally available.

Malaria RDTs are more practical at the point of care in communities where community health workers (CHWs) can be trained in their use, as they do not require electricity or special equipment. RDTs may also detect *Plasmodium* infection even when the parasites are sequestered in the deep vascular compartments and thus undetectable by microscopic examination of a peripheral blood smear. High-quality RDTs have become available [3] and are now the preferred option for programmatic deployment by many national malaria control programs, including Nigeria [6], because of their simplicity and speed in yielding reliable results. Histidine-rich protein II (HRP2)–based RDTs are the preferred options for tropical areas, where *Plasmodium falciparum* is responsible for >95% of malaria infections. In addition, HRP2-based RDTs can withstand better than the enzyme-based RDTs the heat and temperature fluctuations of tropical Africa, where refrigeration and air conditioning are not always feasible. Many have demonstrated their sensitivity, specificity, ease of performance, and reading [7–9].

As part of a large multicenter study conducted in Burkina Faso, Nigeria, and Uganda, evaluating the use of a community-based diagnostic and treatment package for malaria of varying degrees of severity in children aged <5 years in rural communities, we evaluated the performance of an HRP2-based RDT carried out by CHWs. The level of agreement between RDT readings by CHWs vs experienced clinical research staff was also evaluated.

## METHODS

### Study Sites

The studies were conducted in rural communities of Burkina Faso, Nigeria, and Uganda. These sites are described elsewhere in this supplement [10].

#### Burkina Faso

In Burkina Faso, 45 villages were chosen in the health area of Sidéradougou, Mangodara District. The area is a savannah area, with a climate that consists of a rainy season limited to May–October with almost no rain outside this period. This pattern of rainfall defines a very seasonal malaria transmission, with most malaria episodes experienced during or immediately following the rainy season.

#### Nigeria

The Nigerian study was conducted in the Ona-Ara local government area (LGA) of southwest Nigeria between December 2013 and October 2015. The inhabitants are mostly peasant Yoruba farmers and traders. A significant proportion of inhabitants have at least primary school education. Of the 95 villages in 10 wards (2 urban, 2 periurban, and 6 rural) that make up the LGA, 33 villages and 4 of the LGA's 6 rural wards, with an estimated total population of 141 017 and 28 203 children <5 years of age [11], were selected for this study. The climate is that of a tropical rainforest with a warm dry season from November to April and a rainy season from May to October. Malaria transmission occurs year round, with a peak during the rainy-season months and a nadir during the dry-season months.

#### Uganda

Sheema District is located in southwestern Uganda and has a relatively wet climate with annual rainfall of 800–2000 mm, and annual temperature range of 12.5°C–30°C. Sheema is considered to be low transmission for Uganda, but malaria endemicity in the Kayunga area is in-between mesoendemic and holoendemic regions. Transmission occurs throughout the year in both Sheema and Kayunga districts, with peaks associated with the 2 rainy seasons, which are September–January and March–May.

### Study Population (Nigeria)

Children between the ages of 3 and 60 months who presented to CHWs (usually with fever or a recent history of fever suggesting malaria), irrespective of the degree of severity of the illness, were enrolled. Verbal informed consent was obtained from the parents/caregivers of prospective enrollees able to take oral medication, while signed or withnessed verbal informed consent was obtained from the parents/guardians of those unable to take oral drugs (defined as inability to sit, stand, or walk; too weak to eat, drink, or suck; repeated vomiting; altered consciousness or coma; repeated convulsions)—that is, danger signs making them eligible for treatment with rectal artesunate. The history of presenting complaints was obtained and entered into the case report form (CRF) and study register.

### Community Health Workers: Selection, Training, and Procedures in Nigeria

In Nigeria, CHWs were trained on how to obtain informed consent, the signs and symptoms compatible with symptomatic malaria, recognition of danger signs, and theoretical and hands-on performance and reading of malaria RDTs. Training of CHWs also included preparation of thick blood smears on glass microscope slides using aseptic technique and preparation of blood spots for polymerase chain reaction (PCR) analysis and appropriate storage of the various samples [12]. They were also trained on the appropriate dosing of ACTs and the correct method for inserting prereferral rectal artesunate to avoid ejection. Age-specific, color-coded Coartem was purchased from Novartis Pharma agents in Nigeria and rectal artesunate was supplied by WHO, Geneva. The importance of documenting information clearly on the CRF specifically designed for the purpose, while the sick child was still with the CHW, was emphasized. The sick child's details (demographic, symptoms, and findings on testing) were recorded. Initial training was reinforced quarterly during the first year of the study.

### Rapid Diagnostic Test

SD-Bioline malaria *pf* RDT, Lot No. 082365 (Standard Diagnostics, Kyonggi-do, South Korea), an HRP2-based immunochromatographic test which detects HRP2 antigen that is specific for *P. falciparum*, was used in this study. This RDT has very high performance as reported in the most recent annual RDT evaluation program [3] and is approved for public sector procurement by the National Malaria Program of Nigeria [13]. All enrollees were screened for malaria using SD-Bioline RDT following the manufacturer's instructions. The Malaria RDT was considered negative when only the control line was visible, positive when both control and test lines were visible, and invalid when a control line was not seen, irrespective of whether a test line was visible. Test lines were considered present whether faint or deep. Used RDT cassettes were collected at least once in 2 weeks from the CHWs and re-read by a trained research staff with >6 years’ experience reading RDT cassettes. The study cassettes were re-read by the same clinical research assistant and by a senior investigator (C. O. F.) when necessary. All RDTs were from a single batch, stored at the recommended temperature of 4°C–30°C, and were used within shelf life. Malaria RDTs provided to CHWs were replenished at regular short intervals.

### Microscopic Diagnosis

All enrollees in Nigeria had thick blood smears prepared on microscope slides from the same finger prick used for RDTs. In Burkina Faso and Uganda, patients treated with rectal artesunate were normally tested with an RDT and a blood smear at the referral hospital. Dried blood smears were stained with fresh Giemsa stain at pH 7.2 using standard procedures [14, 15]. Stained blood smears were screened in the respective laboratories of the 3 countries for the presence and quantification of malaria parasites using light microscopy at a magnification of ×1000. All blood smears were double-read by 2 experienced malaria microscopists blind to the result of RDTs. Diagnosis of malaria was based on identification of asexual stages of *Plasmodium* on thick blood smears. Parasite density was determined by counting the number of asexual parasites against approximately 200 leukocytes on the thick blood film and converted to parasites per microliter using an assumed total white blood cell count of 8000 cells/µL [14, 15]. Blood films were declared negative if no parasite was seen after viewing 200 high-power fields.

### Clinical Management of Enrollees

Malaria RDT results as read by the CHWs were used to determine management of enrollees. Children who tested positive to RDT without evidence of danger signs received 6-dose Coartem at standard dosage based on age of the children. Children who presented with danger signs received rectal artesunate without waiting for the result of the RDT and were referred to the nearest secondary healthcare facility for definitive management. Children who were referred to health facilities were followed up by the referring CHWs or the study team. Data from the subset of children (155/179 with microscopy) with danger signs from the 3 participating countries are also presented in this article. In addition to the referral, the children who presented with danger signs in Nigeria received financial and sometimes logistic assistance in getting to a secondary healthcare facility and accessing treatment.

### Data Management

Data were entered into EpiData and transferred to Stata software for analysis. With results of microscopy taken as the gold standard, the sensitivity and specificity of the RDT were calculated as TP/(TP + FN) and TN/(TN + FP), respectively, where TP is the number of true positives, TN the number of true negatives, FP the number of false positives, and FN the number of false negatives. Positive predictive values (PPVs) and negative predictive values (NPVs) were calculated as TP/(TP + FP) and TN/(TN + FN), respectively [16]. The level of agreement of RDT readings by CHWs and an experienced expert clinical assistant was evaluated using κ statistics.

### Ethical Approval

Ethical approval for the main study was obtained from the WHO Ethics Review Committee; the University of Ibadan/University College Hospital Institutional Review Committee and the Oyo State Ministry of Health Institutional Review Board in Nigeria; the National Ethics Committee for the Research on Health in Burkina Faso; and the National Council for Science and Technology in Uganda. Individual informed consent was obtained in accordance with the ethical standards set by the different ethics committees.

## RESULTS

There are 2 sets of results presented in this report. The first is parasitology data only from Nigerian children aged 3–60 months assessed by CHWs; the second set is parasitology data from all participating countries (Burkina Faso, Nigeria, and Uganda) for children no longer able to swallow oral drugs and consequently treated with rectal artesunate and referred to the nearest facility.

### Parasitology Data for Nigeria—Uncomplicated Cases

Results of 1860 of 2148 (86.6%) Nigerian children with complete data were analyzed. Parasitology data from 267 children were not available: 20 slides broke, 29 were unreadable, and the identification codes on RDT and microscopy for the remaining 218 enrollees could not be matched. The mean age of the 1860 enrolled children was 30.4 ± 15.7 months (range, 3–64 months) with approximately equal numbers by gender (48.5% males). Details of other patient characteristics are shown in Table 1. In Nigeria, all concomitant symptoms in addition to fever were documented. The most common presenting complaint from enrollees was fever (99.4%), vomiting (30.5%), cough (26.0%), and diarrhea (8.7%). A smaller set of patients had tachypnea (2.5%), and a total of 24 patients were not able to drink or breastfeed (1.3%), had convulsions (1%), were unusually sleepy (0.6%), were unconscious (0.5%), or had edema of both feet (0.4%).

**Table 1.** Characteristics of Febrile Children Presenting With Fever to Community Health Workers in Ona-Ara Local Government Area, Southwest Nigeria

Characteristic	No.	%
Total with QA RDT or microscopy	1860	
Sex		
Male	902	48.5
Female	958	51.5
Age group of enrollees		
<12 mo	257	13.8
12–23 mo	503	27.0
24–35 mo	397	21.3
36–47 mo	422	22.7
48–59 mo	279	15.0
60–72 mo	2	0.1
Clinical presentation all enrollees		
Fever/history of fever	1849	99.4
Vomiting	568	30.5
Cough	483	26.0
Diarrhea	161	8.7
Fast breathing	46	2.5
Not able to drink or breastfeed	24	1.3
Convulsions	19	1.0
Unusually sleepy	12	0.6
Unconscious	10	0.5
Swelling of both feet	8	0.4
MUAC strap		
Red	28	1.5
Yellow	195	10.5
Green	1637	88.0
Malaria parasite detection		
Positive RDTs by CHWs (n = 1860)	1447	77.8
Positive RDTs by expert clinical assistant (n = 1855)	1439	77.6
Positive via microscopy (n = 1593)	862	54.1
Negative microscopy (n = 1593)	731	45.9
Microscopy results not available (n = 1860)	267	14.4

Abbreviations: CHW, community health worker; MUAC, mid upper arm circumference; QA, quality assurance; RDT, rapid diagnostic test.

Malaria parasites were detected year round among the study participants by RDT (Supplementary Figure 1). Prevalence of malaria parasitemia was 77.8% when RDTs were read by CHWs, and 77.6% when the same cassettes were re-read by an experienced clinical assistant (Table 1). Physical examination revealed well-prepared and preserved cassettes. The level of agreement was 86.0% (κ = 0.6; *P* < .001; Table 2). However, the CHWs missed 4 cases of invalid RDT results, all negative on microscopy. One of the 4 was reported as positive while the others were recorded as negative.

**Table 2.** Comparison of Community Health Worker Rapid Diagnostic Test Result Versus Expert Clinical Assistant Results

	RDT Reading by Experienced Clinical Assistance							
	Positive		Negative		Invalid		Total	
RDT by CHW	No.	%	No.	%	No.	%	No.	%
Positive	1312	90.7	132	9.1	2	0.1	1447^a^	100
Negative	127	30.8	284	68.8	2	0.5	413	100
Total	1439	77.4	416	22.4	4	0.2	1860^a^	100

Level of agreement: 86.04% (κ = 0.60, *P* < .001).

Abbreviations: CHW, community health worker; RDT, rapid diagnostic test.

^a^ In the denominators, 1 child with missing reading by expert included.

Eight hundred sixty-two of 1593 enrollees with available results for malaria microscopy had patent parasitemia, giving a parasite prevalence rate of 54.1%. There was no significant difference in the prevalence of malaria parasitemia by age or age group (Table 3). The overall geometric mean parasite density was 6946/µL (range, 40–436 450/µL). The parasite density was between 40 and 200 asexual parasites/µL in 50 patients (5.8%), <500/µL in 106 patients (12.3%), and <1000/µL in 148 (17.2%) of the enrollees with patent parasitemia.

**Table 3.** Details of Malaria Parasite Detection by Microscopy

	RDT Positive			Microscopy-Positive Patients						
	RDT Positive by CHW						Geometric Mean Parasite Density/µL			
	1447	1860		862^a^	1593			Min	Max	
Overall	no.	No.	%	no.	No.	%	6946	40	436 450	*P* Value
Age group										
<12 mo	181	257	70.4	110	224	49.1	8783	40	436 450	.227
12–23 mo	393	503	78.1	250	429	58.3	6479	40	286 560	
24–35 mo	311	397	78.3	177	334	53.0	7813	40	317 200	
36–47 mo	341	422	80.8	196	361	54.3	7623	40	408 000	
48–59 mo	220	279	78.9	128	243	52.7	4734	40	293 600	
60–72 mo	1	2	50.0	1	2	50.0	36 935	36 935	36 935	
Treatment group										
RDT positive by CHW treated with ACTs	1422	1422	100	798	1215	65.7	7707	40	436 450	.013
Rectal artesunate (danger signs)	24	24	100	15	24	62.5	11 428	600	116 560	
RDT negative (CHW) referred, no treatment	0	7	…	0	4	0.0	…	…	…	
RDT negative (CHW) not referred, no treatment	1	407		49^b^	350		1098	40	54 059	

Abbreviations: ACT, artemisinin-based combination therapy; CHW, community health worker; RDT, rapid diagnostic test.

^a^ Parasite density in cumulative percentages: <200 /µL, 5.8%; <500/µL, 12.3%; and <1000/µL, 17.2% patients.

^b^ Ten of 49 (20.4%) and 20 of 49 (40.8%) children who recorded a false-negative RDT result had parasite density >20 000/µL and >3000/µL, respectively.

Table 3 stratifies the study population according to treatment groups using the RDT-positive group treated with ACT as surrogate for uncomplicated malaria and parasite-positive children with danger signs treated with rectal artesunate as children who have severe malaria. Geometric mean parasite density among children with danger signs was significantly higher than among children without danger signs (*P* = 0.013; Table 3). It was noted that 49 of 413 (11.9%) children who tested negative to RDT and in whom microscopy results were available had patent parasitemia with asexual parasites ranging from 40/µL to 54 059/µL. In addition, 10 of 49 (20.4%) and 20 of 49 (40.8%) children who recorded a false-negative RDT result had parasite density above 20 000/µL and 3000/µL, respectively (see Table 3 footnotes). None of the children with a false-negative result presented with danger signs.

The sensitivity and specificity of SD-Bioline RDT as read by CHWs were 94.3% and 41.6%, respectively, while the negative and positive predictive values were 86.1% and 65.6%, respectively (Table 4). Sensitivity and specificity of SD-Bioline RDT results as read by an expert clinical assistant was 92.8% and 40.7%, respectively, while the negative and positive predictive values were 82.7% and 65.0%, respectively. For the more severe group of patients treated with rectal artesunate, sensitivity and specificity of the RDT as read by CHWs were 92.7% and 52.1%, respectively, while the negative and positive predictive values were 92.7% and 52.1%, respectively.

**Table 4.** Overall Performance of Rapid Diagnostic Tests by Community Health Workers Versus Expert Clinical Assistant

Diagnostic Test	Sensitivity	Specificity	PPV	NPV
Children, uncomplicated malaria, with no danger signs				
RDT by CHW	94.3 (92.6–95.8)	41.6 (38.0–45.3)	65.6 (62.8–68.2)	86.1 (82.1–89.6)
RDT reading by expert	92.8 (90.9–94.4)	40.7 (37.1–44.4)	65.0 (62.2–67.6)	82.7 (78.4–86.5)
Children with severe malaria, treated with rectal artesunate				
RDT by CHW	92.7 (80.1–98.5)	52.1 (40.0–63.9)	52.1 (40.0–63.9)	92.7 (80.0–98.5)
RDT reading by expert	92.5 (79.6–98.4)	48.6 (36.4–60.8)	50.7 (38.7–62.6)	91.9 (78.1–98.3)

Data are shown as percentage (95% confidence interval).

Abbreviations: CHW, community health worker; NPV, negative predictive value; PPV, positive predictive value; RDT, rapid diagnostic test.

The prevalence of malaria parasitemia by microscopy was higher during the rainy-season months than during the dry-season months (Supplementary Table 1). In 2014, wet-season compared with dry-season samples were 92% more likely to be malaria positive (odds ratio [OR], 1.92; 95% confidence interval [CI], 1.44–2.47; *P* < .0001,) but there was a nonsignificant 30% risk of positivity for the same period in 2015 (OR, 1.30; 95% CI, .95–1.78; *P* = .107).

### Enrollees With Danger Signs in Burkina Faso, Nigeria, and Uganda

Of the 179 children with danger signs treated with rectal artesunate in all 3 participating countries, at least 2 parasitology readings are available on 166 children: 139 (100%) enrolled in Burkina Faso, 24 (96%) in Nigeria, and 3 (20%) in Uganda. In Burkina Faso, 74.8% of children had no signs of cerebral malaria, whereas in Nigeria the majority (87.5%) who presented for treatment had repeated convulsions, altered consciousness, or coma (Table 5). One hundred eight tested positive at baseline by RDT at presentation to the CHW and 105 tested positive to RDT at the referral hospital. Malaria microscopy was available for 98 enrollees in this subsegment of the study (166 minus 68 where hospital microscopy is not available). The malaria parasite positivity rate by RDT and microscopy among children with danger signs in the 3 countries was 67.9% and 41.8%, respectively. Most patients (94.6% [157/166]) were alive and well at the end of the episode; 7 died and 1 of the enrollees had prolonged hospitalization.

**Table 5.** Clinical Features of Children With Danger Signs Presenting to Community Health Workers in Burkina Faso, Nigeria, and Uganda

Clinical Feature	Burkina Faso n = 139	%	Nigeria n = 24	%	Uganda n = 3	%	Total N = 166	%
Baseline symptoms								
* *No CNS symptoms^a^	104	74.8	3	12.5	2	66.7	109	65.7
* *Cerebral malaria repeated convulsions, altered consciousness, or coma	35	25.2	21	87.5	1	33.3	57	34.3
Malaria parasite detection								
* *RDT positive by CHWs	82	62.1	24	100.0	2	66.7	108	67.9
* *Missing RDT at baseline	7	5.0	0	0.0	0	0.0	7	4.2
* *RDT positive repeated test at referral facility	100	79.4	3	100.0	2	66.7	105	79.5
* *No repeat RDT at referral facility	13	9.4	21	87.5	0	0.0	34	20.5
* *Microscopy positive blood smear, baseline sample	…		15	62.5	…		…	
* *No microscopy at baseline	…		0	0.0	…		…	
* *Positive blood smear, sample at admission, read by expert	28	30.1	…		…		…	
* *No microscopy at admission	46	33.1	…		…		…	
* *Positive blood smear at admission, read by hospital technologist	28	33.3	11	100.0	2	66.7	41	41.8
* *No microscopy at hospital	55	39.6	13	54.2	0	0.0	68	41.0
* *Geometric mean parasite density/µL	38 274		6296		…		24 711	
* *Minimum parasite density/µL	6935		600		…		600	
* *Maximum parasite density/µL	653 395		29 600		…		653 395	
Mean delay in seeking treatment, h								
* *No. with time available	135	97.1	24	100.0	3	100.0	162	97.6
* *Median (IQR)	22.9 (8.2–47.3)		17.5 (3.8–28.0)		20 (6.5–24)		22.2 (8.1–47.0)	
Treatment outcome								
* *Alive and well	134	96.4	20	83.3	3	100.0	157	94.6
* *Alive and hospitalized	0	0.0	2	8.3	0	0.0	2	1.2
* *Died	5	3.6	2	8.3	0	0.0	7	4.2
* *Total	139		24		3		166	

Data are presented as No. (%) unless otherwise indicated.

Abbreviations: CHW, community health worker; CNS, central nervous system; IQR, interquartile range; RDT, rapid diagnostic test.

^a^ Children with repeated vomiting; unable to eat, drink, or suck; prostrated (unable to sit, stand, or walk unaided).

## DISCUSSION

Malaria parasitological diagnosis definitively defines malaria burden and changes in prevalence, targets treatment, supports characterization of treatment response, and enables early identification, investigation, and management of nonmalaria febrile illnesses. This article presents parasitological findings in a study using both RDT and microscopy to guide malaria treatment of uncomplicated and severe episodes requiring either oral ACTs or prereferral treatment with rectal artesunate. Our findings show that when performed by trained CHWs in Nigeria, RDTs are a useful tool for the diagnosis and management of malaria at the point of care in the community. This is consistent with previous findings [17, 18]. There was agreement of 86% betweeen CHW and expert reading of RDT cassettes, which is just average. Because expert re-reading of cassettes took place at the end of the study, some positive RDT results with a faint line might have faded during the interval before re-reading.

We recorded a high sensitivity of 94.3% for SD-Bioline as performed by trained CHWs. However, the specificity of 41.6% obtained is quite low. Findings of high sensitivity and low specificity are not uncommon in areas of high malaria transmission [19, 20]. The most common reason is the fact that HRP2 clears very slowly, with the result that infections that have been cured >2–3 weeks earlier still test positive [21–23].

In the Nigeria study area, which has stable malaria transmission, 34.4% of the children who tested positive to SD-Bioline were free of patent parasitemia and were therefore wrongly diagnosed with malaria and treated with Coartem. This high proportion of false positives could be due to a number of reasons. The persistence of HRP2 from previous infections after cure is well recognized [21–24]. It has been reported that 73% of cases are still positive 35 days after treatment, with the false-positive error associated with initial parasite density [24]; persistence of HRP2 for 14 days after treatment has also been reported despite parasite clearance through microscopy [21]. HRP2 is eventually cleared by anti-HRP2 antibodies, especially anti-HRP2 immunoglobulin G [25]. False-positive RDT results could also be a consequence of the detection limit of microscopy with RDT detecting antigenemia among children with submicroscopic infections. There is also a real possibility that children with recently cured malaria infections seek care for nonmalarial febrile illness; this is not uncommon among children aged <5 years. These nonmalarial fevers could be viral or bacterial.

Although some RDTs are more sensitive than microscopy, there were a significant number of false-negative results associated with RDT compared with microscopy. Quite often, RDT results yield false-negative results when parasite density is low, especially when asexual parasitemia is <200/µL [18] . SD-Bioline is a *P. falciparum–*specific RDT and does not detect *Plasmodium ovale* and *Plasmodium malariae*, which occur in Nigeria, usually as coinfections with *P. falciparum* [26]. In addition, deletion of the HRP2 gene in *P. falciparum* has been reported as a cause of false-negative results with RDTs that are HRP2 based [27, 28]. It is noted that 10 and 20 of 49 children with a false-negative RDT result had parasite densities greater than 20 000/µL and 3000/µL, respectively. This is quite worrisome as all children whose RDT results were negative were denied ACT therapy. The false-negative RDT results despite high parasitemia could have resulted from polymorphism of the HRP2 gene earlier mentioned or from prozone effect, especially those with parasite density >20 000/µL and 1 patient with a parasite density as high as 54 059/µL. Prozone effect, also known as high-dose hook phenomenon, is defined as false-negative or falsely low results in immunological reactions due to an excess of either antigens or antibodies [29]. Deletion of the HRP2 gene of the infecting *P. falciparum* is more likely a rational explanation in these recorded false negatives with parasite density >1000/µL, as prozone effect is usually associated with high parasitemia.

The general performance of SD-Bioline is lower than most previous studies reported from Nigeria and other countries [13, 30]. Ajumobi et al [13] recorded sensitivity, specificity, PPV, and NPV of 100%, 98%, 88%, and 100%, respectively, among symptomatic children aged <5 years in a hospital in a mesoendemic area in Markafi in Kaduna state, Nigeria. The lack of concordance with the study by Ajumobi et al could be related to the difference in study site nature—rural vs semiurban location—as well as the hyperendemicity (54.2% prevalence) in our study vs mesoendemic malaria transmission (10.6% parasite prevalence) in the study of Ajumobi et al [13]. It could also be related to their smaller sample size (295) in a hospital-based study compared with our larger sample size (1500) in a community-based study.

There was a wide gap between malaria parasite detection by HRP2 RDT and microscopy among enrollees who were at the severe end of the spectrum of malaria. The large number of false-positive RDT results is most likely a consequence of delayed clearance of HRP2 following a prior cured infection from the plasma of severely ill children with a nonmalarial cause. This creates a special challenge as microscopy is often not available and even when available and positive, this could be a comorbidity. Aproximately 25% of fatalities in cerebral malaria are found to have other diagnosis at postmortem [31]. The diagnosis of severe malaria among children with danger signs remains a challenge. Quantitative measurement of plasma level of *P. falciparum* HRP2 holds considerable promise in the definitive diagnosis of severe febrile illness in settings of high malaria transmission as an excellent biomarker for cerebral malaria [32].

An important limitation in this study is that children who tested negative to RDT and were denied ACT therapy were not followed up immediately, as blood smears were read much later. PCR assays, which are more sensitive and specific for the diagnosis of malaria, were not done. Finally, expert re-reading of the RDTs was done at the end of the study, with the result that some of the results could have changed within the interval. However, this study indicates that malaria RDTs are useful in guiding malaria treatment of acute uncomplicated malaria in rural Africa despite the higlighted shortcomings of false-negative and false-positive results. The use of combination malaria RDTs (ie, combined HRP2 and pan-malarial parasite specific lactate dehydrogenase-based RDTs) will most likely reduce the challenge of false-negative cases, which is partially responsible for the lack of trust of RDTs among patients and caregivers in Nigeria.

## Supplementary Data

Supplementary materials are available at http://cid.oxfordjournals.org. Consisting of data provided by the author to benefit the reader, the posted materials are not copyedited and are the sole responsibility of the author, so questions or comments should be addressed to the author.

Supplementary Data
