# Supplementary material for: Malaria Rapid Diagnostic Tests and Malaria Microscopy for Guiding Malaria Treatment of Uncomplicated Fevers in Nigeria and Prereferral Cases in 3 African Countries
Source: Clin Infect Dis. 2016 Dec 6;63(Suppl 5):S290–7. doi: 10.1093/cid/ciw628 (PMC5146700; doi:10.1093/cid/ciw628)
Supplement: Supplementary Data [file supp_63_suppl-5_S290__index.html]

Supplementary Data 

# Malaria Rapid Diagnostic Tests and Malaria Microscopy for Guiding Malaria Treatment of Uncomplicated Fevers in Nigeria and Prereferral Cases in 3 African Countries

## Supplementary Data

Supplementary Data

- Supplementary Data - Pdf file
