# Supplementary material for: Malaria Rapid Diagnostic Tests and Malaria Microscopy for Guiding Malaria Treatment of Uncomplicated Fevers in Nigeria and Prereferral Cases in 3 African Countries
Source: Clin Infect Dis. 2016 Dec 6;63(Suppl 5):S290–7. doi: 10.1093/cid/ciw628 (PMC5146700; doi:10.1093/cid/ciw628)
Supplement: Supplementary Data [file supp_ciw628_ciw628supp.pdf]

Supplementary table 1. Malaria parasitaemia by season[Ω]

		Malaria Positivity via microscopy						
		Positive	%	Negative	%	Total	%	Odds Ratio (95% CI) p value
**Season 2014**								
	Rainy	206	58.0	149	42.0	355	100	
	Dry	166	41.8	231	58.2	397	100	1.92 (95% CI 1.44-2.57) p<0.0001
	Total	372	49.5	380	50.5	752	100	
**Season 2015**								
	Rainy	348	64.0	196	36.0	544	100	
	Dry	130	57.8	95	42.2	225	100	1.30 (95% CI .95-1.78) p=0.107
	Total	478	62.2	291	37.8	769	100	

[Ω] A total of 72 patients were enrolled in 2013, and excluded from this table
